# Modulation of the 14-3-3σ/C-RAF “Auto”inhibited Complex by Molecular Glues

**DOI:** 10.1021/jacs.5c12622

**Published:** 2026-01-30

**Authors:** Markella Konstantinidou, Holly R. Vickery, Marloes A. M. Pennings, Johanna M. Virta, Shu Yue Luo, Emira J. Visser, Sean D. Bannier, Mrudula Srikanth, Sabine Z. Cismoski, Lucy C. Young, Maxime C. M. van den Oetelaar, Frank McCormick, Christian Ottmann, Luc Brunsveld, Michelle R. Arkin

**Affiliations:** † Department of Pharmaceutical Chemistry and Small Molecule Discovery Center (SMDC), 8785 University of California San Francisco, San Francisco, California 94143, United States; ‡ Laboratory of Chemical Biology, Department of Biomedical Engineering and Institute for Complex Molecular Systems (ICMS), 3169 Eindhoven University of Technology, Eindhoven 5600 MB, The Netherlands; § Ambagon Therapeutics, de Lismortel 31, Eindhoven 5612AR, The Netherlands; ∥ Helen Diller Family Comprehensive Cancer Center, University of California San Francisco, San Francisco, California 94143, United States

## Abstract

Molecular glues, compounds that bind cooperatively at protein–protein interfaces (PPIs), are revolutionizing chemical biology and drug discovery, allowing the modulation of traditional “undruggable” targets. Here, we focus on a native regulatory PPI between the scaffolding protein 14-3-3 and C-RAF, a key component of the MAPK signaling pathway. Extensive drug discovery efforts have focused on the MAPK pathway due to its central role in oncology and developmental disorders (RASopathies). However, the modulation of its protein complexes is underexplored. C-RAF activity is regulated on multiple levels including dimerization, phosphorylation, and complex formation with 14-3-3, which prevents C-RAF activation by binding to a C-RAF sequence centered on phospho-serine 259. We used a fragment-merging approach to design molecular glues that bound to the composite surface of this 14-3-3/C-RAFpS259 complex. Molecular glues stabilized the inhibitory complex up to 300-fold; their glue-based mechanism of action was confirmed by crystallography and biophysical studies. Selectivity among the other RAF isoforms and other RAF phosphorylation sites was evaluated. The best compounds showed excellent selectivity among a broad panel of 80 14-3-3 clients. Cellular assays demonstrated on-target engagement, enhanced phosphorylation levels of C-RAFpS259, and reduced levels of RAF dimerization and ERK phosphorylation. Overall, this approach enabled chemical biology studies for a C-RAF site that was intrinsically disordered prior to 14-3-3 binding and had not been targeted previously. These molecular glues will be useful chemical probes and starting points for drug discovery efforts to modulate native PPI stabilization in the MAPK pathway with applications in oncology and RASopathies.

## Graphical abstract

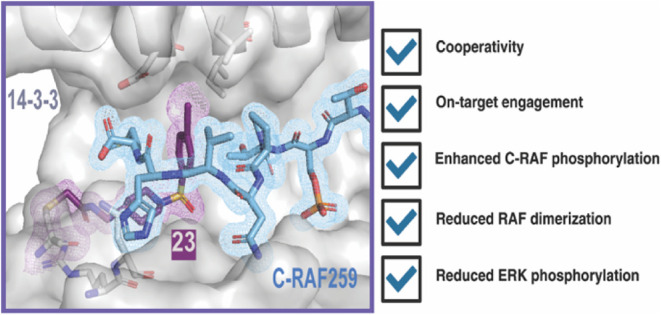

## Introduction

RAF kinases are central regulators of the RAS–RAF–MEK–ERK signaling pathway (MAPK), which controls multiple cellular processes, including cell proliferation, differentiation, and survival.^1^ Frequent mutations, especially in RAS and the B-RAF isoform, lead to aberrant downstream signaling and occur in different types of cancer.^2,3^ Direct inhibitors of components of the MAPK pathway have been the focus of drug discovery efforts for decades.^4,5^

Recently, single-molecule cryo-EM has started to elucidate the structural aspects of MAPK protein–protein interactions (PPIs) as well as the underlying dynamics of pathway activation, providing new opportunities for therapeutic interventions.^6–10^ The regulation of RAF kinases is controlled at different levels^11,12^ by phosphorylation, conformation, dimerization, and binding to 14-3-3 proteins,^13,14^ which are adaptor proteins that recognize specific phospho-serine or phospho-threonine motifs on client proteins.^15,16^ The three RAF isoforms (A-, B-, C-) have highly conserved amino acid sequences and consist of three main domains: the CR1 domain on the N-terminus with the RAS binding domain (RBD) and the cysteine-rich domain (CRD), the CR2 which includes phosphorylation sites, and the C-terminus (CR3), which includes the kinase catalytic domain (CAD).^17,18^ The 14-3-3 binding sites are located in the conserved regions (CRs) on either side of the kinase catalytic domain (Figure 1A). The two 14-3-3 binding sites have opposite functions in the regulation of the pathway; the sites located on CR2 (pS214 for A-RAF, pS365 for B-RAF, pS259 for C-RAF) inhibit RAF activation, while the sites located on the CR3 domain (pS582 for A-RAF, pS729 for B-RAF, pS621 for C-RAF) are activating.^19^

**Figure 1:**
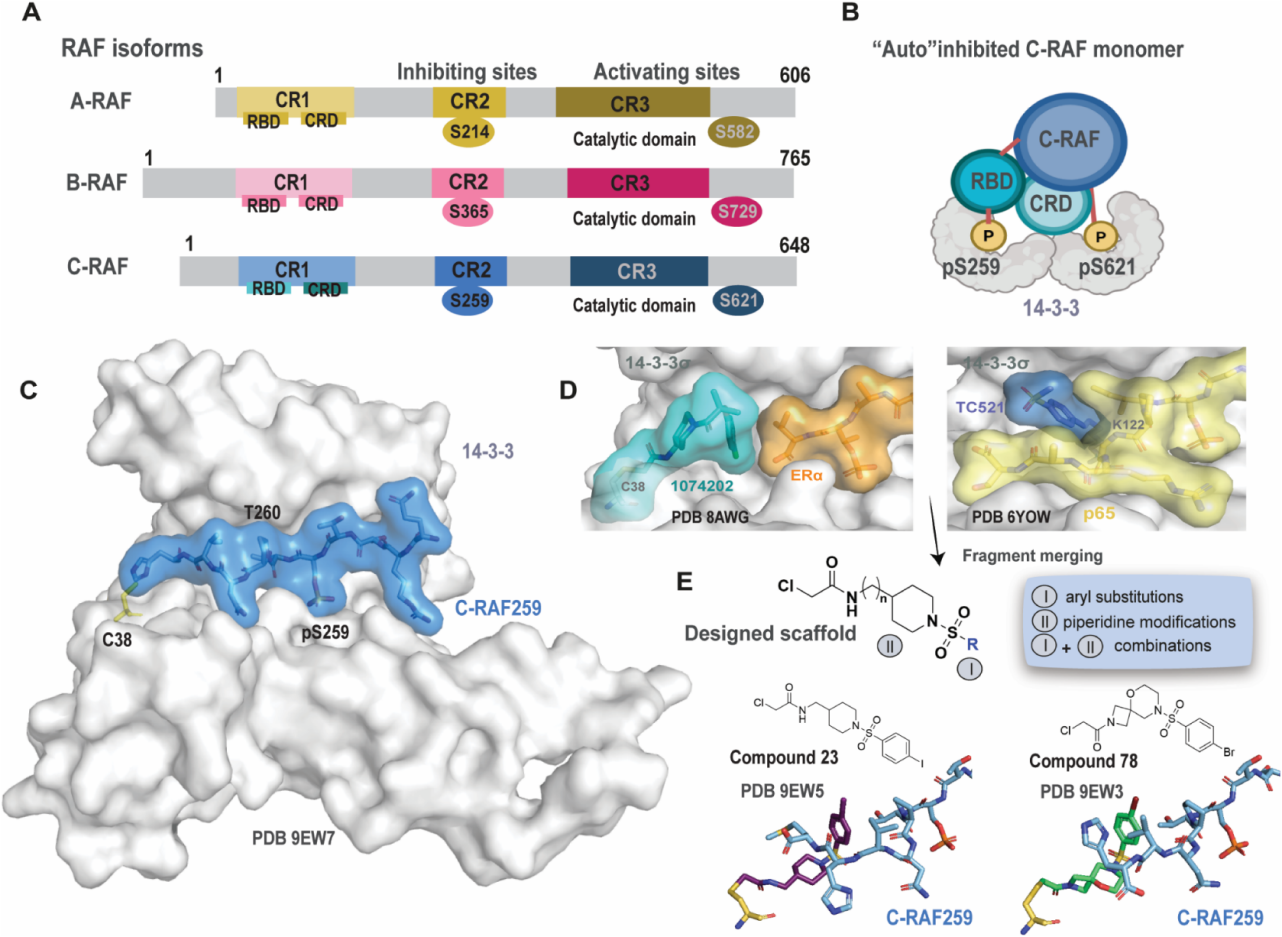
RAF isoforms, 14–3–314-3-3/C-RAF pS259 complex, and fragment merging approach. A) Inhibiting and activating sites on A-, B-, and C-RAF. B) Proposed model for the autoinhibited C-RAF monomer complex bound to a 14-3-3 dimer. C) Crystal structure of 14-3-3σ (gray surface) bound to a phospho-C-RAF259 peptide (cyan surface). C38 (14-3-3) is shown as yellow sticks (PDB: 9EW7). D) Left: Crystal structure of 1074202/14-3-3σ/ERα (PDB: 8AWG). Right: Crystal structure of TC521/14-3-3/p65 (PDB: 6YOW). E) Top: Fragment merging approach toward a designed scaffold for the 14-3-3σ/C-RAF pS259 complex. Bottom: Representative examples and their binding modes (14-3-3 is omitted for clarity; C38 is shown as yellow sticks, and C-RAF as cyan sticks).

In 2019 and 2022, cryo-EM structures of 14-3-3/B-RAF were reported, revealing the activation mechanism of B-RAF.^7,8^ It was hypothesized that C-RAF activation proceeds in a similar manner to B-RAF.^20^ A recent single-molecule FRET (sm-FRET) study on C-RAF supported this hypothesis.^21^ In analogy to the B-RAF cryo-EM structures, in the proposed model, C-RAF is maintained in the autoinhibited, closed state via interactions of the N- and C-terminal domains with a 14-3-3 dimer (Figure 1B). The pS259 site (corresponding to the B-RAF pS365 site) and the pS621 site (corresponding to the B-RAF pS729 site) are each bound in an amphipathic, phosphopeptide-binding groove in the 14-3-3 dimer. Upon pathway activation, C-RAF is recruited to the membrane and interacts with RAS via the RBD domain. This is followed by the release of the CRD domain from the autoinhibited complex and its subsequent interaction with RAS and the membrane. This conformational change exposes the inhibitory C-RAF pS259 site to the phosphatase SHOC2-PP1 for dephosphorylation.^22,23^ RAF is then activated through dimerization of the catalytic domains and stabilized through binding to 14-3-3 via the C-terminal phosphorylation sites only (pS621 for C-RAF and pS729 for B-RAF). Homodimers and heterodimers between all three RAF isoforms are reported.^24,25^

While this paper was under review, cryo-EM structures of the 14-3-3/C-RAF complex were published, showing a similar autoinhibited closed conformation as previously observed with B-RAF.^9^ Additionally, an open monomeric state was resolved, in which the pS259 site and CRD of C-RAF were released from 14-3-3, while pS621 remained bound, exposing the C-RAF dimerization interface. The authors proposed that this open monomer represented an intermediate state between the autoinhibited closed conformation and the active dimer form. Although active C-RAF dimers were not reported in this study, such a configuration was resolved by Dedden et al.,^10^ using an N-terminally truncated C-RAF construct. Collectively, these findings suggested that the activation mechanism of C-RAF is generally similar to that of B-RAF and that the architecture of the autoinhibited closed conformation was consistent in the two RAF isoforms. Nevertheless, notable differences were reported between B-RAF and C-RAF,^9^ with B-RAF preferentially forming homodimers, whereas C-RAF formed heterodimers. Additionally, C-RAF regulation is dependent on HSP90/CDC37, whereas B-RAF regulation is not. C-RAF is less tightly regulated by CRD-mediated autoinhibition compared to B-RAF.

Additionally, distinct roles start to emerge for the RAF isoforms. Recent reports show that C-RAF depletion inhibits tumor growth but not ERK signaling in K-RAS mutant cells,^25^ whereas pan-RAF inhibition affects PI3K signaling in multiple myeloma.^26^ The role of heterodimerization is also an active area of research. The current thinking is that the B-RAF/C-RAF heterodimer is the primary species involved both in native and oncogenic signaling.^27,28^ Notably, the dimerization of C-RAF with A-RAF is also thought to be involved in K-RAS-driven tumor growth^25^ and RAF heterodimers are an important mechanism linked to RAF inhibitor resistance (including “paradoxical activation”) in cancer therapy.^29,30^

Here, our aim is to disrupt the transition from the closed C-RAF monomer to the open, active dimer by stabilizing the autoinhibited 14-3-3/C-RAF pS259 complex (Figure 1C) with molecular glues (MGs)^31^ as an alternative approach to direct C-RAF kinase inhibition. Although direct, selective inhibition of C-RAF has not yet been achieved clinically, a recent study utilizing bio-orthogonal ligand tethering showed that selective C-RAF inhibition promotes paradoxical activation, thus potentially leading to resistance mechanisms.^28^ To date, reported stabilizers of the autoinhibited 14-3-3/C-RAF pS259 complex are the natural product cotylenin-A^32^ (CN-A, Figure S1) and disulfide-tethered fragments identified by our lab.^33^ These disulfide fragments show two distinct binding conformations in crystal structures, although neither appears to make specific contacts with the residues C-terminal to C-RAF pS259 ^33^ (Figure S1). Of note, 14-3-3 has seven highly conserved isoforms. Among them, the sigma isoform has a unique cysteine (C38) at the rim of the 14-3-3/client interface, which is the target for the disulfide fragments. To the best of our knowledge, there is no clear isoform preference for C-RAF binding; hence, the choice of the sigma isoform was based on structural reasons.

Over the past few years, we have used different strategies to develop 14-3-3/client molecular glues. Briefly, for the 14-3-3/ERα complex, we performed structure-based optimization to develop covalent MGs starting from a nonselective disulfide fragment.^34^ Later on, we disclosed a scaffold-hopping approach which resulted in a novel multicomponent reaction-based scaffold.^35^ Additionally, fragment linking was used for noncovalent MGs, starting from cocrystallization of two fragments.^36^ Virtual screening was used to stabilize the 14-3-3/ChREBP complex, an unusual 14-3-3 interaction that lacks a phosphorylated residue.^37,38^ Phosphate- and phosphonate-based compounds were designed and optimized, resulting in cell-active MGs.^38^ We recently reviewed these discovery strategies and techniques used by us and others for 14-3-3/client MGs development.^39^

Here, we used a fragment-merging approach to design molecular glues for the unique composite surface of the 14-3-3/C-RAF259 complex, aiming at specific ligand–protein interactions, including the formation of a covalent bond with C38 (on 14-3-3σ) and polar interactions with T260 (+1 residue on the C-RAF259 phosphorylation site). The most potent stabilizer, compound **23** resulted in 280-fold stabilization of the 14-3-3σ/C-RAF259 complex in fluorescence anisotropy assays, had an EC_50_ of 0.18 μM in a cellular 14-3-3/C-RAF NanoBRET assay and partially blocked C-RAF dimer formation in cells. Compound **78**, although less potent than **23**, showed higher selectivity for C-RAF over the other RAF isoforms in cell assays. Thus, a fragment-based development of MGs provided a tunable selectivity that will help elucidate specific roles of RAF complexes in normal and oncogenic signal transduction.

## Results and Discussion

### SAR Development and Biophysical Assays

Our scaffold design strategy combined a chloroacetamide piperidine moiety (inspired by analog 1074202, a cysteine-targeting 14-3-3/ERα stabilizer^34^) with fragments bearing a sulfonyl group (inspired by TC-521, a lysine-targeting 14-3-3/p65 stabilizer^40^) (Figure 1D). We hypothesized that the sulfonyl group in the new scaffold would serve to induce a pronounced conformational bend in the MG scaffold and act as a flexible handle for potential interactions with T260. Chemical modifications were then performed to optimize the designed scaffold (Figure 1E). Modification (I), in the proximity of the peptide, included optimizing the linkage of the two merged fragments and substitutions on the aryl ring. Modification (II) focused on replacing the piperidine ring with spiro-cycles and fused ring systems with varying sizes and orientations. Lastly, combinations of modifications (I) and (II) aimed to explore synergistic effects.

In analogy to our previous work, two orthogonal assays were used for screening^34^ (Figure S2). Briefly, the mass spectrometry (MS) assay monitored the formation of the covalent bond between electrophiles and the native cysteine (C38) on 14-3-3σ. Dose responses of compounds were performed in the absence and presence of the C-RAF259 phospho-peptide to distinguish between neutral binders and cooperative molecular glues. The experiment was performed as a time course, with measurements every 8 h, after an 1 h incubation. Additionally, the compounds were tested in a fluorescence anisotropy (FA) assay in the presence of the 5-carboxyfluorescein-(FAM)-labeled C-RAF259 peptide. For effective MGs, a significant increase in anisotropy was observed in the overnight measurement. The full dose–response data for all compounds are available in the SI (Tables S2–S5, Figures S5–S8, Figures S15–S18, Figures S25–S32, Figures S37–S39). The dose–response data from both assays were also visually compared in the form of bar graphs. For the MS data, the bar graphs represented the % bound at 1 μΜ compound concentration and 100 nM 14-3-3σ (10:1 ratio) in the absence or presence of the peptide. For FA experiments, the EC_50_ values from the overnight measurement were calculated and plotted as bar graphs showing the positive log EC_50_ value (pEC_50_). Compounds acting as MGs were expected to show increases in both types of bar graphs.

The first key modification of the newly designed scaffold focused on the appropriate linkage of the two merged fragments. Chemically, benzyl or aryl sulfonyl chlorides could be reacted with the appropriate piperidine moiety. Initial screening data from both MS and FA assays showed that benzyl analogs were inactive (compounds **6**–**9**, Figures S3, S5–S7), whereas for phenyl analogs SAR began to emerge as substituents were added on the phenyl ring (Figures S4–S7). Various substituents in the *o*-/*m*-/*p*-positions were added, including electron-withdrawing and electron-donating groups. As a general trend, electron-donating groups were not tolerated, whereas halogens in the *p*-position significantly improved potency, stabilization, and cooperativity (Figure 2A,B, Figures S4–S7, Tables S2–S3). In the MS assay, analogs **11** (*p*-F) and **12** (*p*-Cl) showed binding in the presence of the C-RAF259 peptide and only very low binding in the apo screen (no peptide), indicating cooperative binding. In FA compound titrations, **11** and **12** showed low micromolar EC_50_ values (17 ± 3 and 3 ± 1 μM, respectively) (Table S3, Figure S7). The two compounds were further validated as 14-3-3σ/C-RAF259 molecular glues in FA protein titrations. 14-3-3σ was titrated into 10 nM FAM-labeled C-RAF259 peptide in the presence of DMSO or a saturating concentration of the compounds (100 μΜ). Compounds **11** and **12** decreased the dissociation constant for the 14-3-3σ/C-RAF259 peptide complex by 4-fold and 22-fold, respectively (Figure S8, Table S3). Increasing the linker length of the warhead or replacement of the phenyl ring with a pyridine ring led to loss of potency, and these modifications were not investigated further (compounds **13**–**14**, Figures S4–S7).

**Figure 2:**
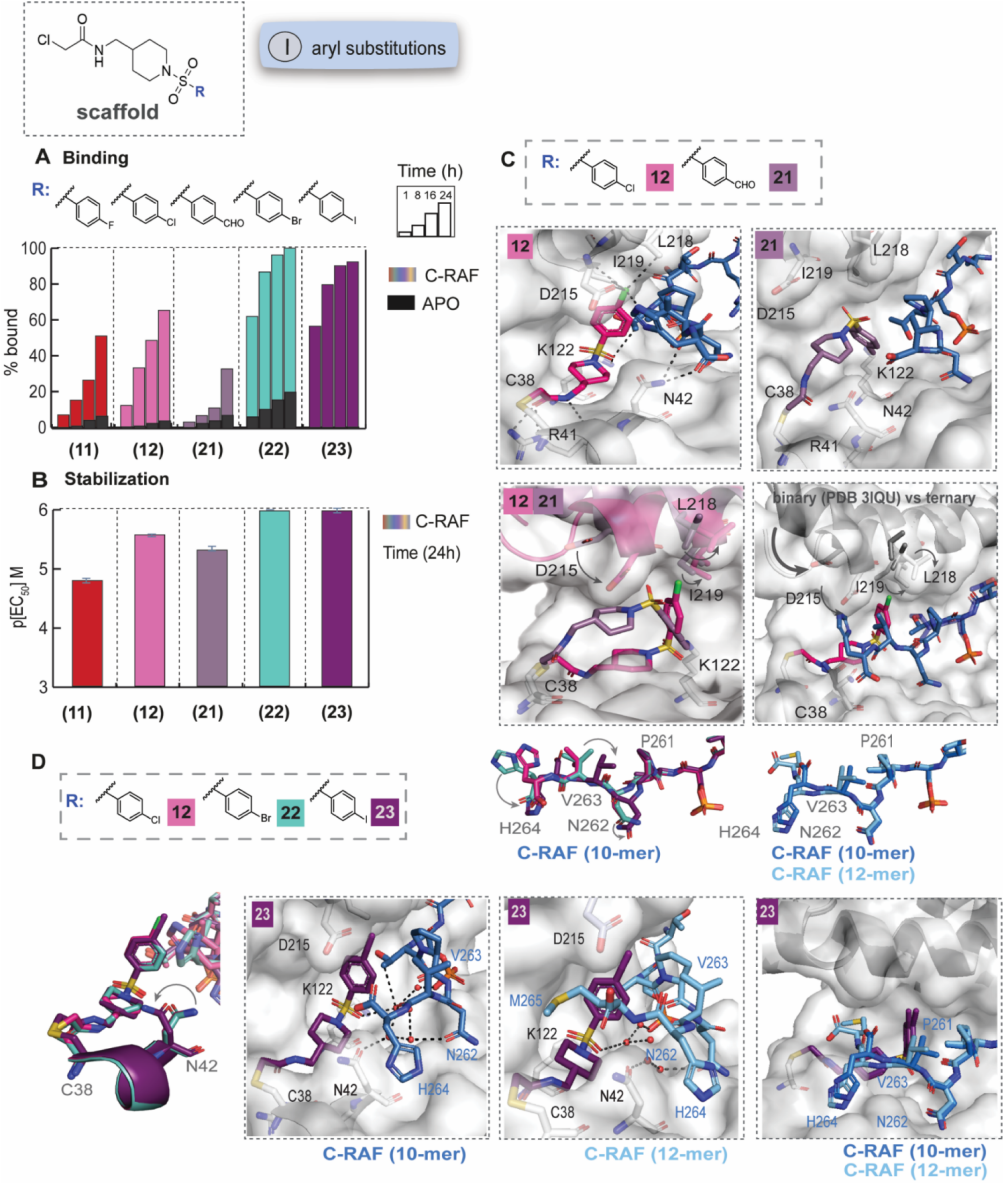
SAR and crystal structures of selected phenyl analogs. A) MS bar graphs at 1 μΜ. For each compound, time course experiments were performed with measurements at 1 h, 8 h, 16 h, and 24 h. C-RAF259 data are shown with different colors for each compound, and apo data in black. B) Bar graphs of FA compound titration pEC_50_ values after overnight incubation. C-RAF259 data are shown with different colors for each compound. C) Top: Crystal structures of **12** (pink sticks) and **21** (light purple sticks) with 14-3-3σ (gray surface) and C-RAF pS259 10-mer peptide (blue sticks). Bottom: Overlays of **12** with **21** (left), and **12** with a binary complex (PDB: 3IQU, right). D) Left: Crystal structures of **12** (pink sticks)/**22** (turquoise sticks)/**23** (purple sticks) with 14-3-3σ and C-RAF259 (overlay). Middle: Detailed interactions of **23**/14-3-3σ/C-RAF259 10-mer (left) and 12-mer (right). Interacting water molecules are shown as red spheres. Right: Overlay of the 10- and 12-mer structures.

To test whether the observed stabilization was unique to halogen substituents or other electron-withdrawing groups could be tolerated, the *p*-formyl analog **21** was synthesized. The two covalent warheads of this analogue could potentially interact simultaneously with C38 and K122 on 14-3-3σ. The analog was weak in the MS assay, and despite the low EC_50_ value (5 ± 1.0 μM) in the FA compound titrations (Figure 2A,B, Figures S4–S7), it resulted in only 9-fold stabilization of the 14-3-3σ/C-RAF259 complex in the FA protein titrations (Figure S8, Table S3). Based on these observations, the presence of halogens seemed to be crucial, and thus bulkier halogens were introduced (*p*-Br (**22**) and *p*-I (**23**)). Both compounds showed faster binding in the MS assay (Figure 2A), and remarkably the *p*-I analog showed almost no binding to 14-3-3 alone; thus, **23** binding was highly cooperative for the 14-3-3σ/C-RAF259 complex. In the FA assay, both compounds showed low micromolar EC_50_ values (1 ± 0.5 μΜ). Consistent with high cooperativity, in protein titrations, the app*K*_d_ of the 14-3-3σ/C-RAF259 decreased from 8427 to 47 nM in the presence of **22** and to 30 nM in the presence of **23**. Thus, **22** and **23** stabilized the 14-3-3σ/C-RAF259 complex by 179- and 280-fold, respectively (Table S3, Figure S8).

Crystal structures of the ternary complexes with 14-3-3σ and a 10-mer C-RAF259 phospho-peptide were solved for **12**, **21**, **22,** and **23** (Figure 2C,D, Figures S9–S10). Overall, **12**, **22**, and **23** formed compact, L-shaped molecules contributing to multiple interactions with both the C-RAF peptide and 14-3-3.

Taking **12** as an example, in addition to the covalent bond with C38 of 14-3-3σ, two hydrogen bonds were formed between the warhead amide, R41 of 14-3-3σ (2.7 Å) and the backbone carbonyl of C38 (3.3 Å). N42 of 14-3-3 directly interacted with the backbone H264 of C-RAF via two hydrogen bonds (3.0 Å). The sulfonyl group of **12** interacted with K122 of 14-3-3 via a charge-assisted hydrogen bond (3.2 Å) and with T260 (+1 position) of C-RAF via a hydrogen bond (3.4 Å).

Based on our past designs^34^ and the conformation of the aldehyde fragment TC521 (Figure 1D), we anticipated that the halogenated/formylated aryl ring would point these substitutions down into a pocket that includes K122 on 14-3-3σ. Instead, **12** (*p*-Cl) adopted a surprising upward conformation at the 14-3-3/peptide interface (Figure 2C, Figure S9) with the halogen pointing into a shallow pocket toward the top of the 14-3-3 binding groove, potentially allowing the formation of halogen bonds with D215, L218, and I219 of 14-3-3 or V263 of C-RAF to increase the stabilization of the complex. In stark contrast, **21** (*p*-CHO) showed a different binding mode, interacting with C38 and K122 via the two covalent warheads. While this conformation more closely mirrored our prediction, no additional interactions were observed with 14-3-3 or C-RAF (Figure 2C). An overlay of the two crystal structures showed a significant conformational change of helix 9 of 14-3-3 for **12** (Figure 2C). The helix moved to a downward position, closer to the compound, thus “clamping” the compound in the 14-3-3 binding groove and causing conformational changes to D215, L218, and I219. An overlay with a previously published binary structure further confirmed that the movement of the helix was compound-induced (Figure 2C).

Crystal structures of **22** (*p*-Br) and **23** (*p*-I) showed an upward binding mode, consistent with **12** (Figure 2D, Figure S10). The key ligand–protein and ligand–peptide interactions were maintained. For **23**, an extensive water network facilitated additional interactions between the compound, N42 (14-3-3) and the C-RAF residues, and extended to the phospho-S259 residue. Interestingly, an overlay of the three structures showed that the C-RAF residues (N262, V263, H264) also adopted different conformations with bigger changes in the case of **23** (Figure 2D, Figure S10).

Notably, the C-RAF259 phosphosite is located on a disordered β-loop , with surrounding C-terminal residues extending well beyond H264 and into the 14-3-3 natural product binding pocket. Although shorter peptides are usually favored for crystallography, we hypothesized that longer peptides might be a better representation of the binding mode for full-length C-RAF. To this end, we solved a binary complex of 14-3-3σ with a 12-mer C-RAF peptide and an additional ternary complex for **23** (Figure 2D, Figure S11). The presence of the compound significantly improved the density observed for the C-RAF sequence and allowed the visualization of M265, which “wrapped” around the main core of the compound, thus significantly covering the binding groove. An overlay of the two ternary complexes for **23** with the 10-mer and 12-mer peptides showed similar conformations for N262, V263, and H264; the latter was oriented toward the outer side of the binding groove in both structures. Consistently, helix 9 adopted a downward conformation in both structures with **23** (Figure 2D).

Structure-guided optimization led to the synthesis of double- and triple-halogenated derivatives to investigate additional electronic and steric effects at the protein–peptide interface (Figure 3A,B, Figure S12, Figures S5–S7). While keeping the electron-withdrawing groups in the *p*-position, we introduced additional F substituents either in the *ortho* or *meta* position. Overall, *m*-F substituents were more favorable than *o*-F. However, in *m*-position bulkier substituents than the F-group significantly decreased both the binding in the MS assay and the stabilization in the FA assay, indicating steric hindrance (Tables S2–S3). Analogs **29** (*m*-F, *p*-Cl), **32** (*m*-F, *p*-CF_3_), and **37** (*m*-F, *p*-Br) were representative examples of the observed SAR (Figure 3A,B, Figure S12). Among them and consistent with previous observations, analog **37** bearing a *p*-Br group showed fast and cooperative binding in the MS assay and an app*K*_d_ of 92 nM in FA protein titrations. Triple-substituted analogs were also tested; however, in protein titrations, they led to lower stabilization compared to **37** (Table S3, Figure S8).

**Figure 3:**
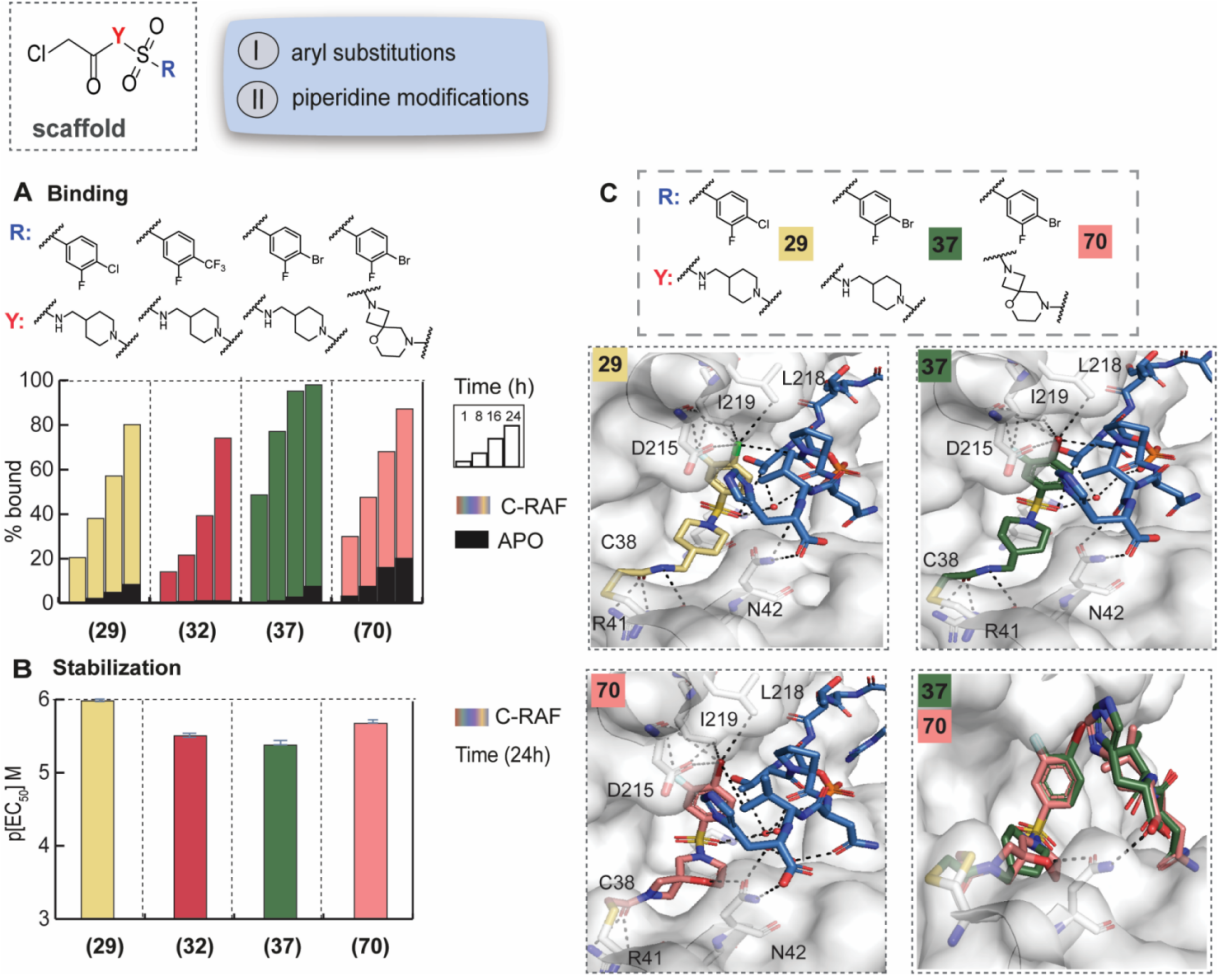
SAR and crystal structures of selected phenyl and spiro analogs. A) MS bar graphs at 1 μΜ. For each compound, time course experiments were performed with measurements at 1 h, 8 h, 16 h, and 24 h. C-RAF259 data are shown with different colors for each compound, and apo data in black. B) Bar graphs of FA compound titration pEC_50_ values after overnight incubation. C-RAF259 data are shown with different colors for each compound. C) Top: Crystal structures of **29** (yellow sticks) and **37** (green sticks) with 14-3-3σ (gray surface) and C-RAF pS259 10-mer peptide (blue sticks). Bottom: Crystal structure of **70** (salmon sticks) with 14-3-3σ/C-RAF259 10-mer and overlay with **37**. Interacting water molecules are shown as red spheres.

Crystal structures with 14-3-3σ and 10-mer C-RAF259 were solved for **29**, **32,** and **37** (Figure 3C, Figure S13). In all cases, the compounds showed similar conformations with helix 9 adopting the downward conformation, as previously observed. The additional *m*-F group was positioned inward and interacted with I219 of 14-3-3 (3.2 Å). This orientation supported the observed SAR, where larger substituents in *m-*position were not tolerated. In *p*-position, the bulkier *p*-Br group formed the most favorable interactions, indicating that the size of the halogens (steric effects), as well as differences in electrostatic effects and σ holes^41^ had a direct effect on the biophysical assays.

Thus, the (*m*-F, *p*-Br)-substitution pattern of **37** was kept constant in the next modification, which focused on the piperidine ring (Figures S14–S18). Substituted piperidines, spiro-cycles, and fused ring systems were investigated. Most of these changes had a negative impact on the potency and, in certain cases, on the cooperativity due to increased apo binding in the absence of C-RAF. One notable exception was analog **70**, with a spiro-ring bearing an oxygen atom. Although in the MS assay it showed less binding than the linear analog **37**, in the FA assay it showed promising stabilization (EC_50_ = 2 μΜ, app*K*_d_ = 58 nM, 95-fold stabilization) (Figure 3A,B, Figures S14–S18). An overlay of the crystal structures of **37** and **70** showed that the spiro-ring was slightly shifted, compared to the piperidine ring, and formed a hydrogen bond with N42 (14-3-3) at 3.0 Å. An extended water network was observed, similar to analog **23**, reaching the phospho-S259 residue (Figures 3C, S19).

Regarding the warhead position, two variations of the chloroacetamide electrophile were tested (structures **74** and **75** in Table S1). Both analogs were completely inactive indicating potentially steric hindrance and unfavorable geometry that prevented access to C38. Warhead modifications were not pursued further; instead we focused our attention on potential synergistic effects by combining favorable aryl ring substituents with spiro-cycles (Figure 4A,B, Figure S20, Figures S15–S18). Overall, analogs **78** and **79**, bearing the same oxo-spiro ring as **70** and *p*-Br and *p*-I substituents, respectively, showed potent stabilization in the FA assay with app*K*_d_ = 35–40 nM (fold-stabilization 210–240, Table S3, Figures S15–S18), but less binding in the MS assay, which correlated with their poor solubility in the assay conditions.

**Figure 4:**
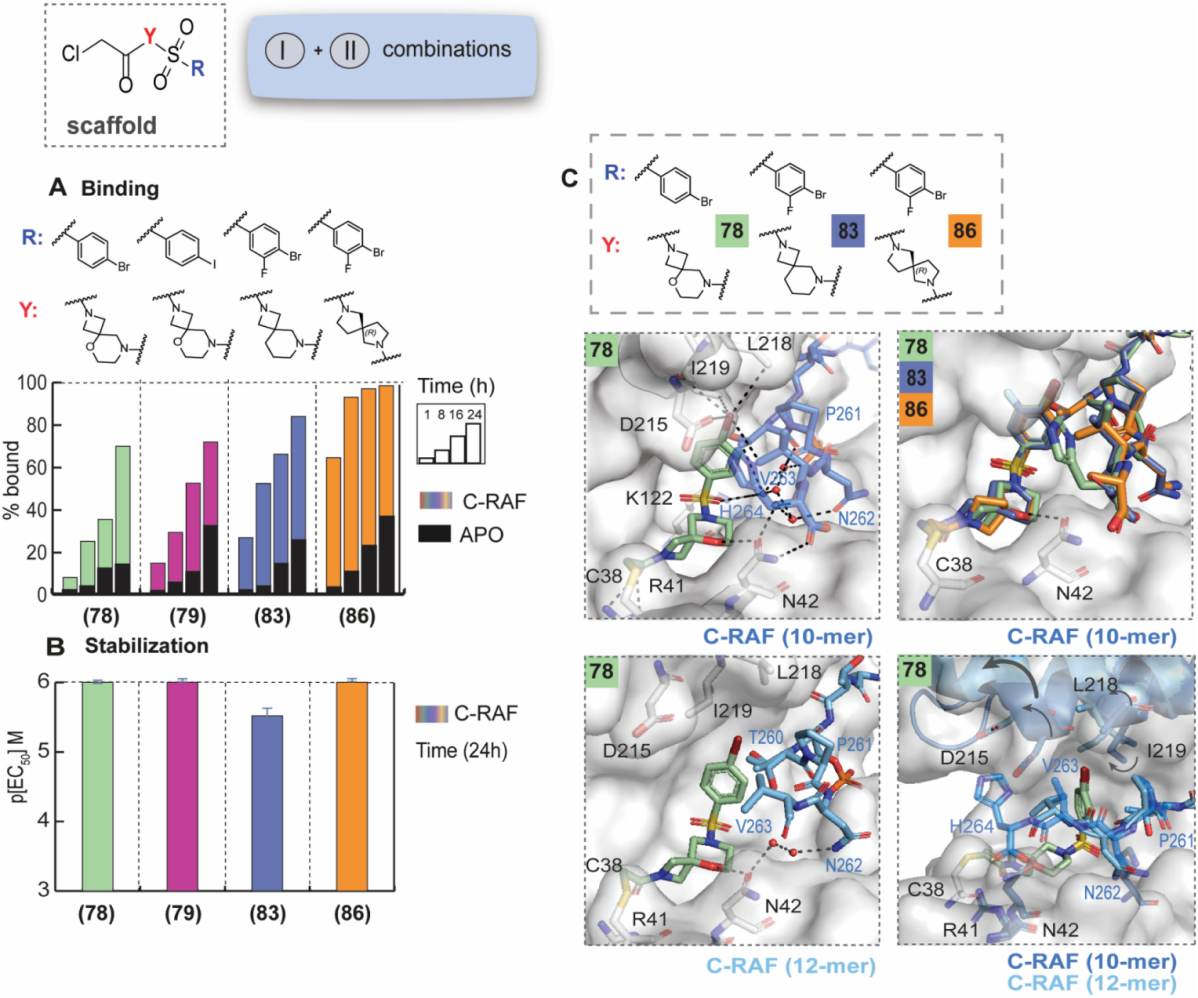
SAR and crystal structures of aryl and spiro/fused rings combinations. A) MS bar graphs at 1 μΜ. For each compound, time course experiments were performed with measurements at 1, 8, 16, and 24 h. C-RAF259 data are shown with different colors for each compound and apo data in black. B) Bar graphs of FA compound titration pEC_50_ values after overnight incubation. C-RAF259 data are shown with different colors for each compound. C) Top: Crystal structure of **78** (light green sticks) with 14-3-3σ/C-RAF259 10-mer and overlay with the **83** and **86** crystal structures. Bottom: Crystal structure of **78** (light green sticks) with 14-3-3σ/C-RAF259 12-mer (left) and overlay of the two ternary complexes (right).

To determine whether the aryl ring or the spiro ring was more important for binding and stabilization, we synthesized analogs **80** and **83**. Replacing the favorable halogen bonds with a *p*-CF_3_ group in analog **80**, while maintaining the spiro-ring dramatically reduced the potency, indicating that the interactions between the aryl ring and V263 (C-RAF) were more significant than the hydrogen bond between the spiro ring and N42 (14-3-3). Similarly, analog **83**, with the same spiro-ring lacking the oxygen, showed slightly reduced potency, compared to **70** (app*K*_d_ = 100 nM, 83-fold stabilization). A pair of enantiomers bearing a 5-membered fused system was also tested (**86**, **89**). The *R*-enantiomer showed more binding in the MS assay and potent stabilization in the FA assay (app*K*_d_ = 28 nM, 300-fold stabilization), in stark contrast to the *S*-enantiomer, which despite binding in the MS assay showed no stabilization in the FA assay.

Crystal structures with 14-3-3σ and 10-mer C-RAF259 were solved for **78**, **79**, **80**, **83**, and **86** and revealed unexpected changes in the interactions with 14-3-3. (Figures 4C, S21). For **78**, **83**, and **86**, helix 9 adopted the downward conformation, “clamping” the compounds and inducing conformational changes in D215, L218, and I219 (14-3-3). However, for **79** and **80**, the helix was in an upward conformation, and thus the compounds lacked these interactions with 14-3-3, while the halogen bond with V263 was not affected. This puzzling effect on helix 9 prompted us to further investigate the binding mode, and we attempted to solve crystal structures with the longer 12-mer C-RAF peptide, which was more challenging. We obtained crystals with **78** and **86**, and in contrast to their binding mode with the shorter peptide, in this case 14-3-3 helix 9 was in the upward conformation. Additionally, less density was visible for C-RAF residues with the longer peptide (V263 instead of H264 for the shorter peptide), hinting potentially at unfavorable steric effects from the spiro-cycle. The overall observations implied that the movement of helix 9 was not entirely compound-induced, but it could also be affected by the length of the peptide used for crystallography. Thus, the significance of some of these interactions needs to be interpreted with caution, since the positioning of helix 9 affects the formation of compound–protein interactions.

To summarize the optimization and SAR, the upward binding mode of the compounds was fundamental for multiple stabilizing interactions of the glues within the 14-3-3/C-RAF259 composite interface. The sulfonyl group made key interactions with K122 (14-3-3) and T260 (C-RAF). The substitutions on the aryl ring had the greatest impact on binding and stabilization with bulky halogens (*p*-Br or *p*-I) leading to the most potent analogs. In *m*-position only a small F substituent was tolerated. The modification of the piperidine ring with spiro-cycles was more challenging due to potential unfavorable steric effects with C-RAF residues. As shown in the crystal structure of **23**, where C-RAF wrapped around the compound, glue–client interactions included the +1 (T260), +4 (V263), and +6 (M265) residues surrounding the aryl ring.

### Selectivity Assessment with Biophysical Assays

After establishing SAR for C-RAF259, we aimed at evaluating potential selectivity: first with the A-RAF and B-RAF inhibiting sites, then with the activating sites, and finally in a broader selectivity panel of diverse 14-3-3 clients. We selected 11 compounds for biophysical assays (**22**, **23**, **29**, **32**, **37**, **70**, **78**, **79**, **80**, **83**, and **86**, and compound **8** as an inactive control, as it lacked an effect on 14-3-3σ/C-RAF259 stabilization).

The sequences of the RAF isoform-inhibiting sites are largely conserved in the regions recognized by 14-3-3; A-RAF214 and C-RAF259 have the same sequence (+1 to +6 amino acids, “TPNVHM” in the C-terminus to pS259), whereas B-RAF365 differs at the +1 position (A instead of T, Figure 5A). The +6/+7 residues for B-RAF also differ compared to A-RAF214 and C-RAF259. More differences occur in the sequences N-terminal to the phospho-serines, which are further away from the compound-binding site and were expected to have a minor effect on the selectivity (detailed peptide sequences in the Methods section in the Supporting Information). Based on the binding mode of the compounds, which interacted with the +1 (T), + 4 (V), and +6 (M) residues of C-RAF, we thus expected similar binding/stabilization to A-RAF and lower binding/stabilization in the case of B-RAF.

**Figure 5:**
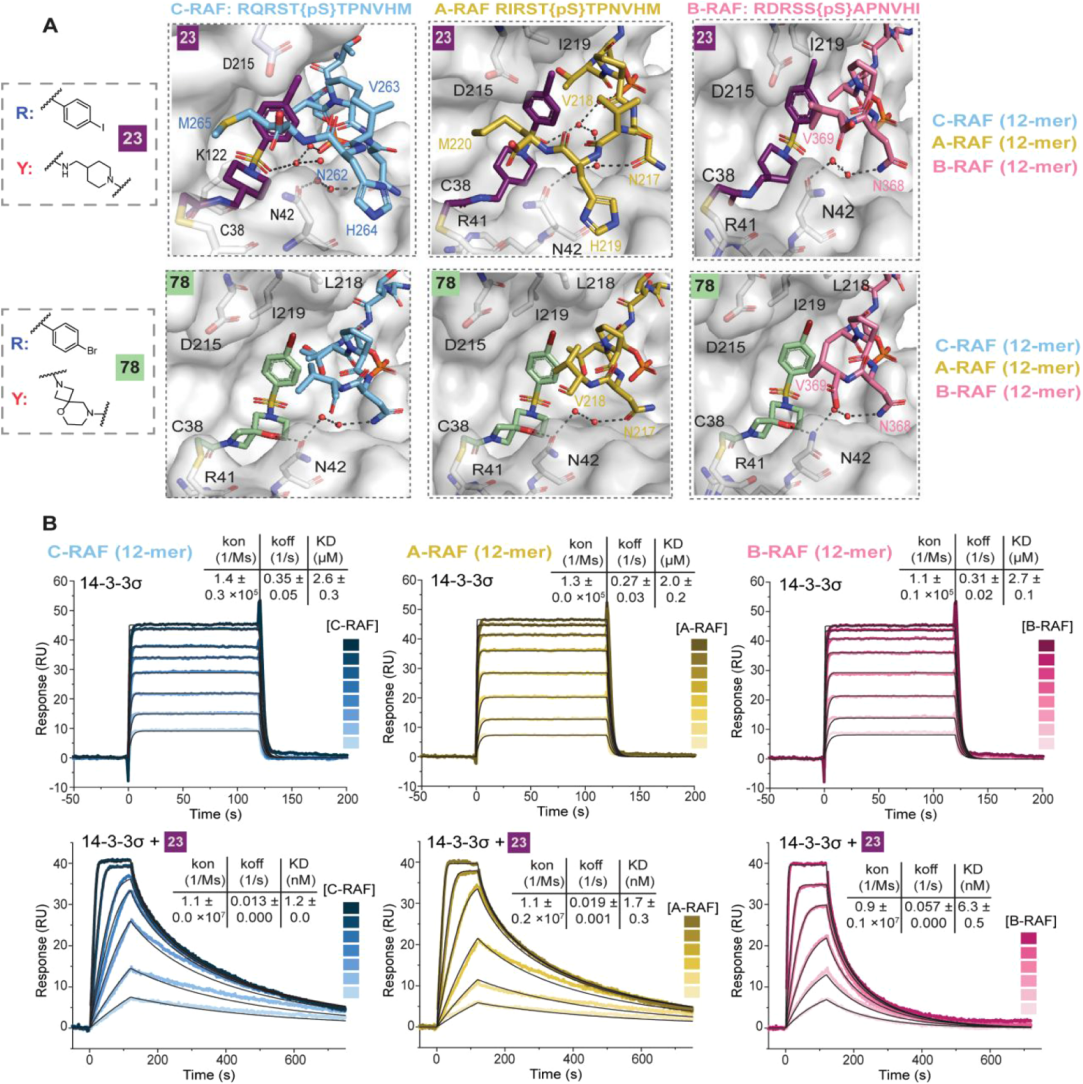
Crystallography and SPR with RAF isoforms. A) Crystal structures of **23** (top) and **78** (bottom) with 14-3-3σ/C-RAF-259 (left), 14-3-3σ/A-RAF214 (middle), and 14-3-3σ/B-RAF365 (right) 12-mer peptides. Interacting water molecules are shown as red spheres. B) SPR data of 2-fold dilutions of C-RAF (blue), A-RAF (yellow), and B-RAF (pink), 12-mer peptides starting at 50 μM binding to 14-3-3σ (top panel), and 14-3-3σ covalently bound to molecular glue **23** (2-fold dilution series of 12-mer RAF peptides starting at 250 nM, parameters shown as mean ± SD, *n* = 2).

Initially we tried to assess selectivity from a structural perspective using 12-mer peptides for crystallography. We solved the binary complexes with 14-3-3 bound to A-RAF214, B-RAF365, and ternary complexes with **22**, **23**, **78**, and **86** for both peptides (Figures 5A, S22–S24) and compared them to the C-RAF259 structures. For the binary complexes, 14-3-3 helix 9 was in the upward conformation, similar to C-RAF259. The observed density for the RAF peptides extended to the +4 residue (V218 for A-RAF214 and V369 for B-RAF365). In the presence of piperidine-containing compounds **22** and **23** with A-RAF214, the +6 M220 residue was visible, and helix 9 was in the downward conformation. For the spiro-cycles **78** and **86**, the visible density did not extend beyond the +4 V218 residue, and helix 9 was in the upward conformation. These observations and interactions in the ternary complexes with A-RAF were similar to those of C-RAF. For B-RAF365, for all four compounds, the visible density stopped at the +4 V369 residue, and helix 9 was in the downward conformation with the exception of **78**. The hydrogen bonds with backbone C38, R41, and N42 (14-3-3) were consistent; however, the compounds lacked specific interactions with the +1 Ala residue, which is significant for molecular recognition of other 14-3-3/client MGs.^33–35,42,43^ As mentioned for C-RAF259, the importance of the movement of helix 9 was ambiguous; therefore, instead we tried to correlate the ligand–protein and ligand–peptide interactions with biophysical data.

14-3-3 generally recognizes between 2 and 20 amino acid residues on the client.^44^ However, the ideal phospho-peptide length, representative of the recognition motif for various 14-3-3 clients, has not been thoroughly investigated. Typically, the +1 residues were expected to be the main driving force for molecular recognition, but in the ternary complexes of our glues with 14-3-3/C-RAF259, the +4 and +6 residues also significantly contributed to binding.

Thus, taking into account the structural differences in C-RAF259 ternary complexes with the 10- and 12-mer peptides, we reconsidered whether this effect might also extend to the biophysical assays and further tested the compounds with peptides of different lengths. Up to this point, to establish the SAR for C-RAF, we used peptides truncated after the +5H residue. For a better comparison with crystallography, we tested the compounds again using 15-mer C-RAF peptides (−7 on the N-terminus, +7 on the C-terminus) before comparing them to A-RAF and B-RAF peptides. In the MS assay with the 15-mer C-RAF peptide, we noticed similar or increased binding for the piperidine-containing compounds (**22**, **23**, **29**, **32**, **37**), but a significant decrease in binding for the spiro-cycles (**78**, **79**, **80**, **83**, **86**) (Table S4, Figure S25). In FA protein titrations, **22** and **23** appeared more potent with app*K*_d_ of 5 nM and 2400-fold stabilization with the 15-mer peptide (30–47 nM, 179–280-fold stabilization with the 10-mer peptide). Compounds **29**, **32,** and **37** showed similar app*K*_d_ values with both peptides (126–154 nM), whereas, in agreement with the MS data, the spiro-cycles (**78**, **79**, **86**) showed 2–3-fold weaker stabilization (app*K*_d_ = 110–334 nM) (Table S5, Figure S26). The data with the longer peptide were thus in support of the crystallography data; for the spiro-cycles, fewer peptide residues were visible compared to compounds like **22**, where the density extended to +6 M and the peptide tightly wrapped around the compound. This observation indicated potential steric hindrance of the longer RAF peptides with the spirocyclic compounds and testified to additional opportunities for MG affinity and selectivity regulation.

To determine compound selectivity, we compared the 15-mer C-RAF data to the 15-mer A-RAF. In the MS assay, the piperidine-containing compounds (**22**, **23**, **29**, **32**, **37**) showed significant binding, comparable to C-RAF, whereas low binding was observed for the spirocycles (Table S4, Figure S27). In FA protein titrations, there were variations in the app*K*_d_ values; however, it was difficult to establish a clear trend (Table S5, Figure S28). We also noticed that although the A-RAF and C-RAF 15-mer peptides had similar *K*_d_ values (8–10 μΜ), the app*K*_d_ in the presence of DMSO differed between A-RAF and C-RAF (3 and 12 μΜ, respectively). This difference did not affect ranking of the compounds with the individual peptides but resulted in higher fold-stabilization values for C-RAF.

In the case of B-RAF365, we tested two different peptides: a 10-mer and a 15-mer, and the results varied considerably, indicating that the 10-mer peptide was probably not a good representation of this PPI (Tables S4 and S5, Figures S29–S30). When comparing the 15-mer B-RAF peptide with A- and C-RAF 15-mer peptides, less binding was observed with the MS assay for the piperidine analogs and consistently low binding for the spiro-compounds (Table S4, Figure S31). A similar effect was observed in the FA assay, where the compounds showed lower stabilization of B-RAF in comparison to the A- and C-RAF 15-mer peptides; **22** and **23** showed app*K*_d_ of 5 nM and 448-fold stabilization; or in other words, they were 3-fold weaker than C-RAF. Consistently, lower fold stabilization was observed with the spiro-cycles (Table S5, Figure S32). These observations were in good agreement with the crystal structures, where the compounds lacked interactions with the +1 residue and less peptide density was visible compared to A- and C-RAF. To summarize, the piperidine analogs demonstrated strong binding and stabilization for C-RAF and A-RAF, whereas the spirocyclic compounds showed significantly lower binding and stabilization across all isoforms, with a slight preference for C-RAF.

The kinetic fundamentals behind RAF isoform selectivity were evaluated using surface plasmon resonance (SPR) experiments. 14-3-3σ, tagged with a Twin-Strep tag, was immobilized on a Strep-Tactin XT-coated SPR chip. Subsequently, a 2-fold dilution series of acetylated 12-mer C-RAF, A-RAF, and B-RAF peptides was injected. The analysis of these peptides’ binary interactions with 14-3-3σ revealed *K*_d_ values ranging between 2.0–2.7 μM, determined through both kinetic- and affinity-based data fitting (Figures 5B, S33). The affinities of A-RAF and B-RAF were consistent with results from FA experiments, while for C-RAF, the *K*_d_ value measured by SPR was lower compared to FA (2.6 μM versus 10 μM, respectively), though this difference might be due to the fast *k*_off_, which makes precise measurement difficult. To compare the kinetic parameters of the binary interactions with those of the molecular glue-bound state of 14-3-3σ, the covalent bond between the chloroacetamide warhead of the compounds and C38 of 14-3-3σ was formed by overnight incubation in the presence of the C-RAF peptide. This resulting complex was immobilized on the chip and extensively washed to remove the C-RAF peptide. Afterwards, the kinetics of RAF peptides binding to the 14-3-3σ/molecular glue complex were analyzed. In the presence of **23**, the association rate (*k*_on_) increased 100-fold for all three RAF isoforms compared to their binary interactions, suggesting that the 14-3-3σ/**23** interface is recognized more rapidly by the RAF peptides than 14-3-3σ alone. Furthermore, *k*_off_ significantly decreased in the presence of **23**, yielding app*K*_d(compound)_ values in the low nanomolar range (Figures 5B, S34–S36). Notably, the presence of the molecular glue resulted in mass-transport limitations, driven by the high association rate and peptide rebinding. These effects were accounted for during the fitting process (Table S6). The affinity increase was most pronounced for A-RAF and C-RAF (1000-fold and 2000-fold, respectively), whereas a weaker increase (400-fold) was observed for B-RAF, aligned with the selectivity measured in FA experiments. Since the shift in *k*_on_ was similar for all RAF isoforms, the difference in affinity among isoforms was attributed to a faster *k*_off_ for B-RAF compared to A-RAF and C-RAF. This indicated that the interactions of +1 and +6 residues (+1 A and +6 I for B-RAF, +1 T and +6 M for A-RAF and C-RAF) with **23** were crucial for decreasing the *k*_off_, thereby increasing the residence time of RAF binding to 14-3-3σ. In addition, **22** induced a comparable change in *k*_on_ for A-RAF and C-RAF binding, relative to **23**. However, the *k*_off_ value was increased, leading to a reduced apparent affinity, in line with FA experiments. While no notable difference was observed between **22** and **23** for B-RAF, the apparent affinity of B-RAF to 14-3-3/**22** complex remained the weakest among the RAF isoforms (Figures S34–S36).

Regarding the activating A-RAF582, B-RAF729, and C-RAF621 sites, all isoforms contain the same +1 to +4 residues (EPSL). The +1 glutamic acid, in a published binary complex with C-RAF621, formed an ionic bond with K122 of 14-3-3 (PDB: 4IEA). We hypothesized that this bond could not be disrupted by the compounds and would also prevent the compounds from binding due to repulsive electrostatic interactions with the sulfonyl group of the compounds. This hypothesis was easily confirmed with FA protein titrations, where no stabilization was observed (Figures S37–S39).

To evaluate selectivity more broadly *in vitro*, we tested **22** and **23** in a selectivity panel that included 80 peptides representing a diverse set of 14-3-3 clients (Figure S40). Briefly, the 14-3-3σ protein concentration was set at 20% of the maximum effect for each individual peptide. The compounds were incubated overnight at calculated EC_90_ concentrations (under this assay’s conditions: 10.5 μM for **22** and **23**) in the presence of 14-3-3 and 100 nM client peptides. A client peptide with a high 14-3-3σ concentration (50 μM, maximum effect) was used as a reference for normalization. As a qualitative output, clients were classified as hits when a positive change in anisotropy of more than 15 units (ca. 15%) was observed. Both compounds showed remarkable selectivity; for **23** only four clients emerged as hits (SOS1, TAZ, KC1A, TSC2), and six for **22** (SOS1, TAZ, ARGH2, H31, KC1A, TSC2). Strikingly, there were no apparent similarities in the peptide sequence for these off-target hits, except for the +2 P, which is a very common residue in 14-3-3 clients (Figure S41A). The lack of similarities in the peptide sequences suggested that conformational adaptivity was a key factor in the formation of ternary complexes and molecular recognition among different 14-3-3 clients. FA compound titrations were then performed for the common hits SOS1, TAZ, KC1A, and TSC2 (Table S7, Figure S41B) and FA protein titrations were performed for SOS1, TAZ, and TSC2 (Table S8, Figure S41C). The assay conditions and the C-RAF259 peptide length differed from those used to establish the SAR, leading to an EC_50_ = 5 μM (vs 1 μM routinely seen with the standard conditions). Nevertheless, the compounds showed greater fold-stabilization and lower EC_50_ values for C-RAF, with the second-most prominent hit being TAZ.

### Evaluation in Cell Assays

Expressing and phosphorylating full-length C-RAF are highly challenging due to its low expression levels and tendency to aggregate during purification. In recently published cryo-EM structures of the 14-3-3/C-RAF complex, the authors introduced specifc B-RAF residues into the C-RAF sequence to improve protein stability.^9^ Therefore, instead of trying to express and purify full-length C-RAF, we investigated the effects of compounds within a cellular context. Ten compounds were included in cellular assays; the same compounds tested for RAF isoform selectivity, excluding the weak analog **80**. Compound **8**, as previously mentioned, served as a negative control.

A NanoBRET assay was developed to measure the binding of full-length 14-3-3σ to C-RAF in HEK293T cells.^45^ HEK293T cells only weakly express 14–3–3σ and show relatively low levels of pathway activity under normal growth conditions; thus, the transfected C-RAF and 14-3-3σ could be monitored without interference from endogenous expression. C-RAF was tagged with NanoLuciferase and 14-3-3σ was tagged with a HaloTag/fluorescent HaloTag ligand. Stabilization of the interaction with a molecular glue was expected to lead to an increase in the intensity of the BRET signal. The tested compounds resulted in a range of increased fold-stabilization (up to 1.6) and cellular EC_50_ values between 0.1 and 1.2 μΜ (Figure S42A, Table S9). Piperidine-containing compounds **22** and **23** showed similar EC_50_ values (0.20 and 0.18 μΜ, respectively) and fold stabilization of 1.5 and 1.6-fold, respectively (Figure 6A). For the spirocycles **70** and **78,** cellular fold stabilization was lower (1.3 and 1.4, respectively), in good agreement with the signal window observed in FA data. No increase in the BRET signal was observed for negative control compound **8**. The stabilization effect was specific to C38 of the σ isoform of 14-3-3 —the molecular glues did not increase the BRET signal when the native C38 was mutated to N, the residue found in the other 14-3-3 isoforms (Figure S42B).

**Figure 6:**
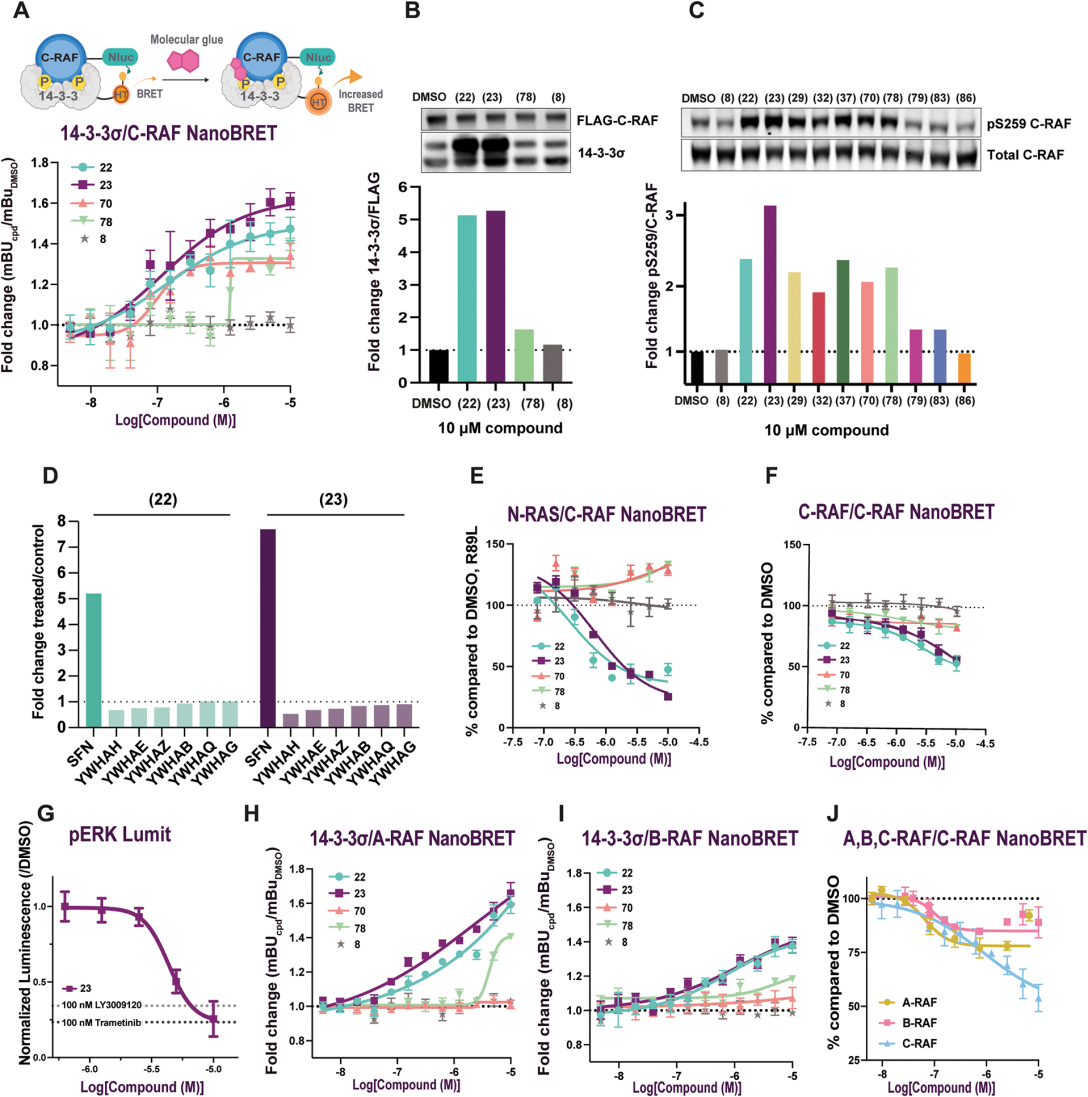
Evaluation of molecular glues in cell assays. A) 14-3-3σ-Halotag (HT)/C-RAF-NanoLuciferase (Nluc) NanoBRET schematic (top) and dose–response curves in HEK293T cells. B) Western blot and quantification of co-IP of FLAG-C-RAF and HA-14-3-3σ transfected HEK293T cells after treatment with 10 μM compound overnight using anti-FLAG magnetic beads. C) Protection of phosphorylation of the C-RAF pS259 site in HEK293T cells transfected with FLAG-C-RAF and HA-14-3-3σ. Overnight treatment was performed with 10 μM compound. D) Anti-C-RAF IP-MS in NCI-H226 cells treated with DMSO or 10 μM of **22** or **23** for 24 h. C-RAF bound all seven 14-3-3 isoforms. 14-3-3σ (SFN) significantly increased the abundance (**22**/DMSO: FC = 5.2, *p*-value = 0.020; **23**/DMSO: FC = 7.7, *p*-value = 7.9 × 10^–04^). E) N-RAS/C-RAF NanoBRET; 100% was considered the DMSO mBU and 0% the mBU value of NRAS/C-RAF R89L, which does not bind to C-RAF. F) C-RAF/C-RAF homodimers NanoBRET dose–response curves; 100% was considered the DMSO mBU. G) Lumit immunoassay for pERK levels in dose–responses with **23** compared to RAF inhibitor (LY3009120) and MEK inhibitor (Trametinib). H) 14-3-3σ/A-RAF NanoBRET. I) 14-3-3σ/B-RAF NanoBRET. J) NanoBRET for heterodimer formation for A-RAF/C-RAF and B-RAF/C-RAF, compared to C-RAF homodimers NanoBRET for **23**.

Binding of full-length 14-3-3σ/C-RAF was further validated in HEK293T cells through coimmunoprecipitation (co-IP) at 10 μΜ compound. N-RAS Q61L, FLAG-C-RAF, and HA-14-3-3σ were transfected, and IP was performed with anti-FLAG beads. We used N-RAS Q61L, an oncogenic mutation, to induce pathway activation, which included reduced C-RAFpS259 and increased binding to N-RAS.^46,47^ We measured a 5.3-fold increase for **23**, 5.1-fold for **22,** and 1.6-fold for **78** in a 14-3-3σ pull-down with FLAG-C-RAF normalized to FLAG band intensity (Figure 6B). This effect was not observed without transfecting C-RAF, as shown for **22** (Figure S43A).

In addition to binding assays, we quantified the effect of the molecular glues on a 14-3-3-specific function—the protection of phosphorylated residues on the client proteins from phosphatases (Figure 6C, Table S9). By Western blots in HEK293T cells, with 10 μΜ compound we measured 2.4-fold and 3.2-fold increases in phosphorylation of the C-RAF259 site relative to total C-RAF for **22** and **23**, respectively. The spirocycles **70** and **78** showed a slightly smaller effect (2.1-fold and 2.2-fold, respectively). In MIA PaCa-2 cells, a KRAS G12C-dependent cell line, compounds **22** and **23** showed a 1.4-fold and 1.5-fold increase in endogenous C-RAF pS259 levels relative to total C-RAF (Figure S43B, Table S9). To test whether this effect was specific to the stabilization of the 14-3-3σ/C-RAF complex, we performed a Western blot with the 14-3-3 C38N mutant. As expected, no increase in the pS259 levels was observed (Figure S43C). In addition, we immunoprecipitated endogenous C-RAF in NCI-H226 cells and characterized changes in the C-RAF interactome using mass spectrometry. Consistent with the proposed mechanism, we found that 14-3-3σ (SFN) was the only 14-3-3 isoform that showed increased binding to C-RAF under treatment with **22** or **23**, with 5.2-fold and 7.7-fold increases, respectively (Figure 6D).

As mentioned in the introduction, the activation mechanism of C-RAF is assumed to proceed in a similar manner to B-RAF (based on the cryo-EM structures), starting with the recruitment of the autoinhibited RAF monomer to the membrane through ionic interactions between RAS and the exposed basic residues of the RBD.^8,48^ Formation of the ionic bonds with RAS would generate steric clashes and electrostatic repulsion at the RBD:14-3-3 interface that would initiate a rearrangement in 14-3-3 dimer binding, resulting in the disassembly of the autoinhibited monomer and the exposure of additional RBD residues involved in full RAS–RBD contact. Our hypothesis was that stabilization of the 14-3-3/C-RAF autoinhibited complex with MGs would prevent some of the flexibility required to get full/stable RAS–RBD contact, since the CRD plays a central role in maintaining RAF in the autoinhibited state through interactions with the kinase domain and both 14-3-3 protomers. To study the effect of the molecular glues on this step of the pathway, we developed a NanoBRET assay to measure the interaction between N-RAS Q61L and C-RAF in HEK293T cells (Figure 6E). For the quantification of the N-RAS/C-RAF NanoBRET, we set the DMSO-treated cell signal as 100% and the signal for N-RAS/C-RAF R89L, a C-RAF mutation that prevented C-RAF from interacting with RAS, as the 0% control.^30,49^ The stabilization of the interaction between 14-3-3 and C-RAF259 by the molecular glues was expected to compete with the interaction of N-RAS/C-RAF. As expected, treatment with **23** resulted in 75% reduction of the N-RAS Q61L/C-RAF interaction and 52% with **22**. For **70** and **78**, no clear trend was observed. Compound **8** did not affect the N-RAS Q61L/C-RAF binding.

The final activation step of the pathway includes the dimerization of the RAF kinase domains. Dimer formation is quite complex, and different RAF homo- and heterodimers can occur depending on the cell type or cancer type. We aimed to study potential effects of the molecular glues on RAF-dimer formation using a NanoBRET assay. The C-RAF/C-RAF NanoBRET assays were performed with N-RAS Q61L to ensure pathway activation^50^ (Figure 6F). We observed a 43% decrease in C-RAF homodimer formation with **22**, 47% decrease with **23**, and ∼20% for **70** and **78**.

To test the downstream MAPK effects of stabilizing 14-3-3/C-RAF, we developed a Lumit immunoassay to analyze the levels of pERK. The secondary antibody pair, lysis buffer, and primary antibody concentration were optimized for the ERK/pERK antibodies used (data not shown). 14-3-3σ and C-RAF were transfected in a 1:1 ratio in HEK293T cells. Cells were treated with **23**, a MEK inhibitor (Trametinib, 100 nM), or a pan-RAF inhibitor (LY3009120, 100 nM) in 0.5% FBS FluoroBrite DMEM for 24 h, followed by EGF stimulation (100 ng/μL, 30 min) before performing the Lumit immunoassay protocol. We corrected for cell number using the fluorogenic live-cell GF-AFC substrate. Data were normalized to DMSO-treated samples. Compound **23** dosed at 10 μM inhibited the phosphorylation of ERK to a similar extent as the MEK inhibitor (Trametinib) and slightly better than the pan-RAF inhibitor (LY3009120); the IC_50_ value for **23** was 4.4 μM (Figure 6G). No effect was observed for the negative control compound **8** (Figure S44A) or with the C38N mutant upon treatment with **23**. Of note, Trametinib showed the same effect with 14-3-3σ and 14-3-3 C38N (Figure S44B). Thus, the Lumit data suggested that stabilization of 14-3-3/C-RAF was an effective mechanism to modulate the MAPK pathway.

We developed additional NanoBRET assays to quantify compound binding to 14-3-3σ/A-RAF and 14-3-3σ/B-RAF and determined RAF selectivity in a cellular context (Figure 6H,I, Figure S45, Table S10). Compounds **22** and **23** showed binding in the 14-3-3σ/A-RAF NanoBRET, giving 1.6-fold and 1.7-fold increases, respectively, at 10 μM; dose–response data did not plateau. **70** was inactive, and **78** showed an effect only at the two highest compound concentrations. Similarly, in the 14-3-3σ/B-RAF NanoBRET, increased binding was observed in the presence of **22** and **23** (1.40-fold increase) and a minor effect was observed for **70** and **78**. Quantitatively, **22** and **23** showed comparable fold-stabilization for A- and C-RAF, whereas **70** and **78** showed significant selectivity for C-RAF, especially at lower compound concentrations. In all cases, binding was dependent on C38, as the compounds were inactive with the C38N mutant (data not shown). We also tested the formation of C-RAF heterodimers A-RAF/C-RAF and B-RAF/C-RAF. Compound **23** exhibited inhibition of heterodimers, decreasing the A-RAF/C-RAF interaction by 23% and the B-RAF/C-RAF dimer by 15% (Figure 6J).

The compound effect on the phosphorylation protection of the inhibiting A-RAF pS214 site was also quantified and compared to the effect on the C-RAF pS259 site (Figure S46, Table S10). Compound **23** resulted in a 1.3-fold increase in the case of A-RAF and a 3.2-fold increase in the case of C-RAF. Compound **70** did not increase the A-RAF phosphorylation, whereas it showed a 2.1-fold increase for C-RAF. A similar experiment was performed for the B-RAF pS365 site; however, the quantification of the Western blots was inconsistent, potentially due to the antibody used (data not shown).

Taken together, the cell data suggest on-target activity on the 14-3-3σ/C-RAF259 complex for the most potent compounds **22** and **23**, a protective effect on C-RAF259 phosphorylation, disruption of C-RAF active kinase dimers, and a decrease in phosphorylation of the downstream MAPK target ERK. Regarding SAR, the presence of a piperidine ring was more favorable in cell assays compared to the spirocycles, supporting our crystal structures, where interactions occur with M265 (+6 residue). Interestingly, while the piperidine-containing **22** and **23** favored C-RAF over A- and B-RAFs, the spirocycles showed an even stronger selectivity for C-RAF.

## Conclusions

In summary, we describe a fragment-merging approach for the development of cell-active molecular glues targeting the inhibitory 14-3-3σ/C-RAF259 complex. Validation of the compounds in multiple biophysical assays, from mass spectrometry to fluorescence anisotropy and surface plasmon resonance, allowed the quantification of binding, stabilization, and kinetics, elucidating the factors involved in the formation of cooperative ternary complexes. The establishment of SAR for the 14-3-3σ/C-RAF259 complex was achieved with biophysical assays using phosphopeptides as representations of the full-length client, since protein expression and purification of phosphorylated C-RAF are notoriously challenging. Overall, the molecular glues showed selective binding to C38 of the 14-3-3σ isoform, observed as single labeling in the intact MS assay, and further proven by crystallography of ternary complexes. MS and FA assays were in good agreement in establishing SAR for a given peptide length for C-RAF259. However, we found that the longer C-RAF peptides led to differentiated results, especially in the case of the spirocyclic compounds, where the rigid part of the compound was in close proximity to the flexible region of the peptides. These effects were not exclusively steric based on space-filling models; rather, we hypothesized that entropy and/or less optimal interactions with the +4 and +6 peptide residues were also involved. The peptide length also had a strong effect on compound evaluation for B-RAF, indicating again that residues involved in molecular recognition can extend well beyond the +1—here, as far as the +4 and +6 residues, offering enhanced potential for selective compound design. By comparing data using peptides of different lengths, we aimed to emphasize that for 14-3-3 clients where the phosphorylation site lies on a highly dynamic loop, conformational adaptivity can have a significant effect on both the biophysical assays and crystallography. Thus, in such cases, the peptide length should be chosen carefully to accurately mimic the full-length protein.

From crystal structures with different RAF peptide lengths, we observed differences not only in the peptide conformation, which was expected, but also in the positioning of helix 9 of 14-3-3. The movement was not always compound- or peptide-specific, but it could have a significant effect on the formed ligand–protein interactions. It is noteworthy that the downward positioning of this helix is consistent with cryo-EM structures of 14-3-3/B-RAF and C-RAF (PDB: 6NYB, 9MMP), indicating that it is unlikely to be a crystallographic observation. An overlay of the cryo-EM and crystal structures revealed comparable binding of the RAF phosphorylation sites within the 14-3-3 pocket (RMSD of 0.670 Å for C-RAF and 0.847 Å for B-RAF alignments) (Figures S47, S48). The cryo-EM structure showed unresolved regions around the C-RAF pS259 site, specifically before residue R254 (−5 R) and after H264 (+5 H), comparable to those seen in the binary crystal structure. In the presence of compounds **22** and **23**, additional electron density corresponding to residue M265 (+6 M) became visible, indicating stabilization of disordered regions in full-length C-RAF (Figure S47).

We further evaluated the 10 most potent compounds from the biophysical assays in cell assays with full-length RAF clients. We noticed a good potency correlation in most of the cases. Compounds **22** and **23** were the most potent in cell assays and stabilized both the 14-3-3/C-RAF and 14-3-3/A-RAF complexes, whereas spirocycles **70** and **78** showed greater selectivity for C-RAF in the NanoBRET assay and the protection of phosphorylation. Taken together, our cell data indicate that the molecular glues showed on-target activity, stabilizing the 14-3-3σ/C-RAF interaction and protecting the pS259 site from dephosphorylation. Of note, these effects were specific to the 14-3-3σ isoform and the presence of C38, as no effect was observed with the C38N mutant. Similarly, the IP-MS data further proved that the other 14-3-3 isoforms, which contain cysteine residues in different positions, were not affected by our molecular glues. Mechanistically, we see the expected inhibition of the N-RAS/C-RAF interaction as well as C-RAF dimerization in an activated pathway for the most potent analogs. These data indicate that the molecular glues targeted the autoinhibited 14-3-3/C-RAF monomer, thus shifting the equilibrium away from the active form of C-RAF. The effect on the structurally similar A-RAF isoform was chemotype-dependent, with piperidine compounds **22** and **23** being less selective compared to spirocycles **70** and **78**. The observed differences in A-RAF stabilization are intriguing, considering the sequence similarity with C-RAF on the 14-3-3 binding groove. Possible explanations could be differences in the N-terminus, dynamics, or regulation at a cellular level. The similarity of the A-RAF isoform could explain the observed inhibition of A-RAF/C-RAF complexes with **23,** whereas the B-RAF/C-RAF dimer was minimally affected.

Quantifying the downstream effects of stabilizing the 14-3-3/C-RAF complex, **23** prevented the phosphorylation of ERK to levels similar to those of other modes of MAPK modulation. However, **23** was slower to modulate the pathway than Trametinib or LY3009120, taking 18 to 24 h compared to 3 h. We hypothesize that the time delay is due to the slow reaction of the covalent warhead rather than the intrinsic biology of 14-3-3/C-RAF stabilization. The kinetics do complicate the biological analysis since the MAPK pathway includes multiple feedback loops and varied dimer formation. These might explain why we do not see full inhibition of C-RAF dimerization, nor do we observe on-target cell death in cancer cells such as MIA PaCa-2. Park et al.^51^ solved the cryo-EM structure of K-RAS and MEK bound to 14-3-3/B-RAF, in which B-RAF pS365 (the equivalent to C-RAF pS259) is still phosphorylated and bound to 14-3-3. Interestingly, our data indicate that 14-3-3/C-RAF stabilization can significantly inhibit binding to RAS, suggesting that multiple mechanisms of RAF activation (and inhibition) are possible.

Additionally, recent literature suggests that C-RAF has roles independent of the MAPK signaling pathway, which we have not explored. For instance, Venkatanarayan et al. showed that C-RAF depletion inhibits tumor growth but not ERK signaling in KRAS mutant cells, and upon C-RAF loss, A-RAF dimers promote ERK signaling, leading to cell-cycle arrest.^25^ Sanclemente et al. reported that in KRASG12V/Trp p53 mutant lung tumors, systematic abrogation of C-RAF expression did not inhibit canonical MAPK signaling which, in contrast to MEK inhibitors, resulted in limited toxicities.^52^ For these types of tumors, significant levels of phospho-MEK and phospho-ERK expression were retained, despite the elimination of C-RAF expression in most of their cells.^52^ Alternatively, the C-RAF preference of our glues may limit phenotypic changes. Müller et al. showed that selective knockdown of a single RAF isoform had at best partial effects on the phosphorylation levels of MEK1/2 and ERK1/2 indicating functional redundancy and/or compensatory mechanisms between the RAF isoforms, while pan-RAF activity affected PI3K-dependent signaling.^26^ Deciphering the precise effect of these molecular glues on C-RAF biology and broader pathway changes is beyond the scope of the current study. We believe, however, that our molecular glues will be useful chemical biology tools that extend beyond direct inhibition and focus on the regulation of protein–protein interactions. Additionally, our cell-active compounds could be useful starting points for medicinal chemistry campaigns aiming at either pan-RAF/14-3-3 glues or RAF-isoform-specific glues to further elucidate pathway biology.

## Supplementary Material





## Notes

(XLSX)
Experimental methods, supplementary figures and tables, synthetic procedures, compound characterization, NMR spectra, and crystallography data (PDF)
